# Effects of Myostatin on Nuclear Morphology at the Myotendinous Junction

**DOI:** 10.3390/ijms24076634

**Published:** 2023-04-02

**Authors:** Hikari Amemiya, Masahito Yamamoto, Kazunari Higa, Genji Watanabe, Shuichiro Taniguchi, Kei Kitamura, Juhee Jeong, Nobuaki Yanagisawa, Ken-ichi Fukuda, Shinichi Abe

**Affiliations:** 1 Division of Special Needs Dentistry and Orofacial Pain, Department of Oral Health and Clinical Science, Tokyo Dental College, 2-9-18 Kandamisaki-cho, Chiyoda-ku, Tokyo 101-0061, Japan; kitamurahikari@tdc.ac.jp (H.A.); kfukuda@tdc.ac.jp (K.-i.F.); 2 Department of Anatomy, Tokyo Dental College, 2-9-18 Kandamisaki-cho, Chiyoda-ku, Tokyo 101-0061, Japan; yamamotomasahito@tdc.ac.jp (M.Y.); watanabegenji@tdc.ac.jp (G.W.); taniguchisyuuichirou@tdc.ac.jp (S.T.); 3 Ophthalmology/Cornea Center, Tokyo Dental College Ichikawa General Hospital, 5-11-13 Sugano, Ichikawa, Chiba 272-8513, Japan; higakazunari@tdc.ac.jp; 4 Department of Histology and Developmental Biology, Tokyo Dental College, 2-9-18 Kandamisaki-cho, Chiyoda-ku, Tokyo 101-0061, Japan; kitamurakei@tdc.ac.jp; 5 Department of Basic Science and Craniofacial Biology, New York University College of Dentistry, 345 E. 24th Street, New York, NY 10010, USA; 6 Division of Oral Health Sciences, Department of Health Sciences, School of Health and Social Services, Saitama Prefectural University, 820 Sannomia, Koshigaya-shi, Saitama 343-0036, Japan; yanagisawa-nobuaki@spu.ac.jp

**Keywords:** myostatin, Achilles tendon injury, C2C12, NIH3T3, myotendinous junction

## Abstract

Myostatin (Myo) is known to suppress skeletal muscle growth, and was recently reported to control tendon homeostasis. The purpose of the present study was to investigate the regulatory involvement of Myo in the myotendinous junction (MTJ) in vivo and in vitro. After Achilles tendon injury in mice, we identified unexpected cell accumulation on the tendon side of the MTJ. At postoperative day 7 (POD7), the nuclei had an egg-like profile, whereas at POD28 they were spindle-shaped. The aspect ratio of nuclei on the tendon side of the MTJ differed significantly between POD7 and POD28 (*p* = 4.67 × 10^−34^). We then investigated Myo expression in the injured Achilles tendon. At the MTJ, Myo expression was significantly increased at POD28 relative to POD7 (*p* = 0.0309). To investigate the action of Myo in vitro, we then prepared laminated sheets of myoblasts (C2C12) and fibroblasts (NIH3T3) (a pseudo MTJ model). Myo did not affect the expression of Pax7 and desmin (markers of muscle development), scleraxis and temonodulin (markers of tendon development), or Sox9 (a common marker of muscle and tendon development) in the cell sheets. However, Myo changed the nuclear morphology of scleraxis-positive cells arrayed at the boundary between the myoblast sheet and the fibroblast sheet (aspect ratio of the cell nuclei, myostatin(+) vs. myostatin(-): *p* = 0.000134). Myo may strengthen the connection at the MTJ in the initial stages of growth and wound healing.

## 1. Introduction

The myotendinous junction (MTJ) is a complex anatomical structure where skeletal muscle connects to tendon. This connection has an integral role in transmitting muscle contractile force to tendon fibers. The collagen fibers in tendon are anchored to the cell membrane surrounding a skeletal muscle fiber, the sarcolemma [1]. This cell membrane is folded into finger-like processes to increase the area of muscle–tendon contact [1]. At the molecular level, the MTJ consists of two independent transmembrane linkage systems, the dystrophin-associated glycoprotein complex (DGC) and α7β1 integrin [1]. These systems link muscle cells to the tendon extracellular matrix (ECM) via laminin 211 [2]. Recently, proteomics and single-nucleus RNA-seq studies of skeletal muscle have identified a marker protein of the MTJ, collagen XXII [3]. Collagen XXII is present on the muscle side of the MTJ, whereas collagen XIV and XII are expressed on the tendon side [4].

The development and regeneration of the MTJ remains an active area of research. The axial muscle and tendon originate from distinct somatic elements, i.e., the myotome and sclerotome, respectively [5]. Despite their different origins, the two are connected through several steps [1]. At the first step, the myoblasts are located close to tenocytes, which secrete an extracellular matrix (ECM). At the second step, a newly formed basement membrane can be identified at the interface between the myotubes and tenocytes. Finally, sarcolemmal resistance to increasing contractile forces intensifies, along with progressive parallel alignment of the tendon collagen fibers. At the molecular level, desmin, a muscle-specific type III intermediate filament protein, accumulates at the tendon side of the muscle [6,7]. Fibroblast growth factor 4 (Fgf4) expressed at the end of the muscle induces tenascin-C and scleraxis in the tendon [8]. Recently, Esteves et al. [9] and Yaseen et al. [10] have reported that lateral plate-derived fibroblast-to-myoblast conversion contributes to morphogenesis of the MTJ. However, unlike reports describing the development of the MTJ, only a few studies have focused on its regeneration. Yan et al. [11] have identified muscle–tendon progenitor cells that have the capacity to regenerate the MTJ. These progenitor cells co-expressing muscle and tendon markers are located in the MTJ. In humans and mice, many adipocytes are located close to the MTJ, and may promote its active repair [4].

Myostatin (Myo), or growth differentiation factor 8 (GDF-8), is a 26 kDa glycoprotein belonging to the TGF-β superfamily. Myo is primarily synthesized in skeletal muscle, suppressing its growth [12,13]. Recent studies have reported that Myo is involved in the maintenance of tendon morphology [14]. Myo knockout reduces the number of fibroblasts in tendons, thereby reducing overall tendon size [14]. Aside from results obtained using Myo-deficient mouse models, increased muscle mass and a 20% decrease in maximum tendon strain have been similarly observed in rats. Functionally, however, tendon peak strain decreases, even if peak stress and stiffness remain unchanged [15]. As detailed above, the effects of Myo on tendon tissues have been studied from various perspectives, but the effects of Myo at the tenocyte developmental stage, located preferentially at the tips of tendons close to muscles (MTJ), have not been fully clarified.

C2C12 is a myoblast cell line derived from mouse striated muscle satellite cells [16]. C2C12 can form muscle fibers under appropriate stimulation and is used as a model of in vitro muscle regeneration [16]. Moreover, C2C12 is a multipotent progenitor cell with the ability to differentiate into not only muscle but also osteoblasts and adipocytes [17]. Treatment of C2C12 with bone morphogenetic protein 2 results in a typical osteoblast phenotype [18], while thiazolidinediones and fatty acids induce C2C12 differentiation into adipocytes [19]. In recent years, lamination of C2C12 cells on a scaffold with fibroblast growth factor-2 has been shown to induce differentiation into tenocytes [19]. Furthermore, Myo can induce C2C12 differentiation into tendons via SMAD family member 3 (Smad3) [20].

NIH3T3 is a fibroblast cell line that was originally isolated from a mouse NIH/Swiss embryo [21]. As they maintain expression of human telomerase reverse transcriptase, NIH3T3 can be employed as feeder cells [22]. The co-culture of cells with NIH3T3 stimulates their growth. For example, corneal epithelial cell sheets co-cultured with NIH3T3 are useful for regeneration of the ocular surface [23], and an NIH3T3 double feeder system is useful for engineering corneal epithelial sheets [24]. Three-dimensional (3D) co-culture with NIH3T3 maintains the liver function of hepatocyte spheroids [25]. NIH3T3 cells are associated with limb mesodermal tissues, which include muscle and tendon [26].

In this context, we suspected that Myo might alter cell morphology to aid recovery and development of the MTJ after experimental observation injury. Recently, a novel MTJ model was established using a co-culture of C2C12 myoblasts with NIH3T3 fibroblasts on a collagen fiber scaffold [27]. Therefore, co-culture of these two cell lines would be a useful approach for investigating regeneration and development of the MTJ. In the present study, we were able to show that lamination of NIH3T3 on C2C12 promoted tenogenic differentiation in the superficial layer of a myoblast cell sheet, and that Myo altered the nuclear morphology of the tenoblast-like cells in this layer, suggesting the role of Myo in MTJ formation.

## 2. Results

### 2.1. Regeneration Processes on the Tendon Side of the MTJ

**Figure 1:**
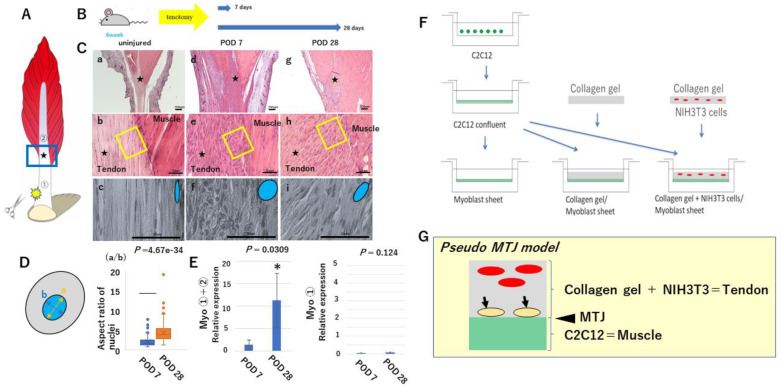
Schematic presentation of the experimental work. MTJ development in mouse embryos. (**Panel A**) Schematic drawing of the gastrocnemius muscle and its tendon. Achilles tendon injury in mice. (**Panel B**) The mice are euthanatized with CO_2_ on postoperative days 7 and 28 (POD7 and POD28). (**Panel C**) The processes of tendon regeneration at the MTJ. (Panels **a**,**d**,**g**) low-magnification view, (Panels **b**,**e**,**h**) medium-magnification view, (Panels **c**,**f**,**i**) high-magnification view. Squares in Panels **b**,**e**,**h** correspond to Panels **c**,**f**,**i**. Stars indicate the same location. Tendon cells are elongated fibroblast-like cells (Panels **a**–**c**). Unexpected cell accumulation on the tendon side of the MTJ (Panels **d**–**i**). These cells were more numerous at POD7 than at POD28 (Panels **e**,**f**,**h**,**i**). (**Panel D**) The aspect ratio of nuclei on the tendon side of the MTJ differed significantly between POD7 and POD28 (POD7: 2.13 ± 1.13, POD28: 4.33 ± 2.29, N = 4). (**Panel E**) Myostatin mRNA expression in the two groups. Although in ① Myo was rarely expressed on POD7, in ① + ② Myo expression was significantly increased on POD28 (POD7: 1.39 ± 1.00, POD28: 11.24 ± 6.09, N = 4). * *p* <0.05. (**Panel F**) Preparation of three types of laminated sheets and morphological observation: C2C12, C2C12 laminated with collagen gel (C2C12-Gel), and a myoblast sheet laminated with collagen gel mixed with NIH3T3 (C2C12-Gel 3T3). (**Panel G**) Schematic drawing of the pseudo MTJ model (arrow: S cells).

Tendon cells appeared as elongated fibroblast-like cells with cytoplasm that was stretched between the collagen fibers of the tendon. Each had a central spindle-shaped nucleus (Figure 1C(a–c)). After Achilles tendon injury in mice (Figure 1A,B), unexpected cell accumulation was noted on the tendon side of the MTJ (Figure 1C(e,f,h,i)). These cells were more numerous at POD7 than at POD28 (Figure 1C(d,e,g,h)). Morphologically, nuclei at POD7 had an egg-like profile, whereas those at POD28 had a spindle shape (Figure 1C(f,i)). The aspect ratio of nuclei on the tendon side of the MTJ differed significantly between POD7 and POD28 (Figure 1D) (*p* = 4.67 × 10^−34^). We investigated the expression of Myo mRNA in the injured Achilles tendon in mice (Figure 1A,E). We performed real-time PCR for Myo, and compared its expression at POD7 and 28 at two locations: ① near the site of Achilles tendon injury, and ① + ② near the site of injury and distant from it (i.e., Achilles tendon + intramuscular tendons + muscle) (Figure 1A). At site ① Myo was rarely expressed at POD7 and 28 (*p* = 0.124) (Figure 1E). However, at sites ① + ②, Myo expression was significantly increased at POD28 relative to POD7 (*p* = 0.0309) (Figure 1E). Therefore, it appeared that Myo was involved in tendon regeneration at the MTJ, being expressed at the end of the muscle to induce tendon differentiation [8].

### 2.2. Developmental Processes on the Tendon Side of the MTJ

**Figure 2:**
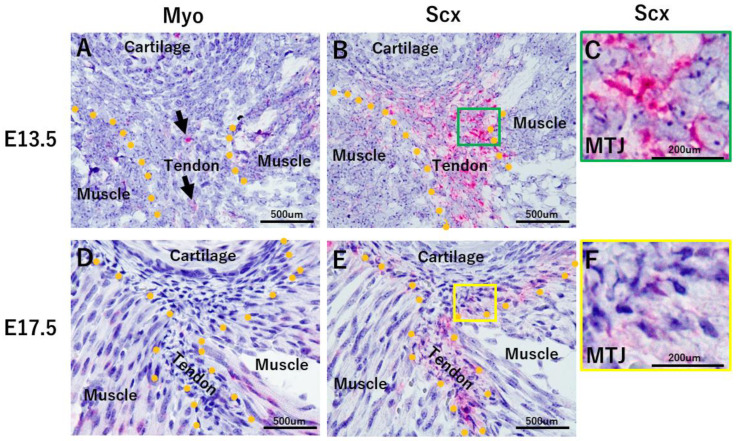
On embryonic day (E) 13.5, Myo is expressed in myoblast cells and a few tenoblast cells (two arrows) (**Panel A**). Tenoblast cells clearly express Scx (**Panel B**). On E17.5, myotube expresses Myo (**Panel D**). Tendon shows Scx expression (**Panel E**). There are numerous Scx-positive cells exhibiting a round nuclear morphology (**Panel C**). On E17.5, many Scx- positive cells exhibit spindle-shaped nuclei (**Panel F**). E13.5: *n*= 3, E17.5: *n* = 3. Squares in Panels **B**,**E** correspond to Panels **C**,**F**.

On embryonic day (E) 13.5, Myo was expressed in myoblast cells and a few tenoblast cells (Figure 2A). Tenoblast cells clearly expressed Scx (Figure 2B). On E17.5, Myotube expressed Myo (Figure 2D), while tendon cells showed Scx expression (Figure 2E). There were numerous Scx-positive cells exhibiting round nuclear morphology (Figure 2C). However, on E17.5, many Scx-positive cells exhibited spindle-shaped nuclei, and cells with a round nucleus were rarely observed (Figure 2E).

### 2.3. Preparation of the Pseudo MTJ model (Part 1)

**Figure 3:**
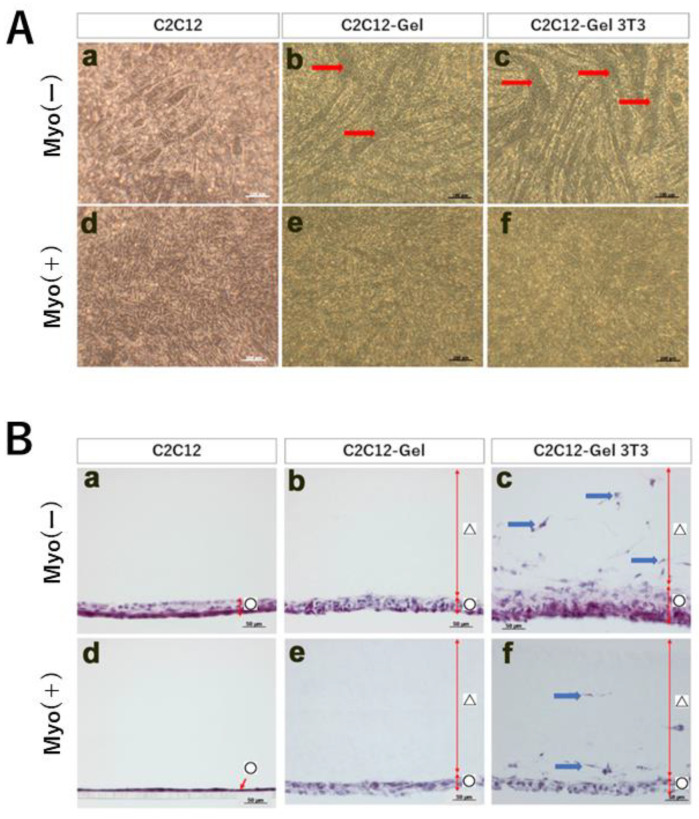
Preparation of the pseudo MTJ model after 1 week of culture (Part 1). (**Panel A**) Morphological observation of laminated sheets. Three types of cell sheets (C2C12, C2C12-Gel, and C2C12-Gel 3T3) were cultured without Myo(−) and with myostatin Myo(+), resulting in A: Observation in a bright field. Compared to C2C12 (**a**), myotube structures are observed in C2C12-Gel (**b**) and C2C12-Gel 3T3 (**c**) (red arrows). In Myo(+), no myotube-like structure was observed in any type of cell sheet (**d**–**f**); (**Panel B**) Hematoxylin–eosin staining of frozen thin sections. In comparison to C2C12 (**a**) and C2C12-Gel (**b**), cell sheets are thicker in C2C12-Gel 3T3 (**c**). Addition of Myo did not increase the thickness of any type of sheet (**d**–**f**). ○: Myoblast sheet; △: Collagen gel; Blue arow: cells present in collagen gel overlayed with gel-3T3.

For this MTJ model, we prepared three types of cell sheets: ① C2C12, ② C2C12-gel, and ③ C2C12-Gel 3T3 (Figure 1F) [28,29]. These sheet types were divided into Myo-free (Myo(−)) and Myo (Myo(+)) groups, and cultured for a week (Figure 3). In Myo(−) cultures, myotube formation, a marker of muscle maturation, was observed in C2C12-Gel (Figure 3A(b)). In addition, myotube formation was more advanced in C2C12-Gel 3T3 sheets (Figure 3A(c)). However, in C2C12 sheets, no myotube formation was observed (Figure 3A(a)). Furthermore, in Myo(+), no myotube formation was observed in any type of cell sheets, suggesting that myostatin suppressed muscle maturation (Figure 3A(d–f)). Next, to observe the thickness of the three types of cell sheets, we prepared thin sections and performed hematoxylin–eosin staining (Figure 3B). In Myo(−) cultures, cell sheet thickness was increased to a greater degree in C2C12-Gel 3T3 than in C2C12 and C2C12-Gel (Figure 3B(a–c)). However, in Myo(+) cultures, cell sheet thickness did not increase in C2C12-Gel 3T3 (Figure 3C(d–f)). These results showed that lamination of collagen gel on myoblast sheets aids myoblast maturation, and that NIH3T3 further enhances this tendency. As it has been reported that Myo mutation leads to an increase in the proportion of muscle progenitors present within muscles [30], the addition of Myo may suppress muscle maturation.

### 2.4. Preparation of a Pseudo MTJ Model (Part 2)

**Figure 4:**
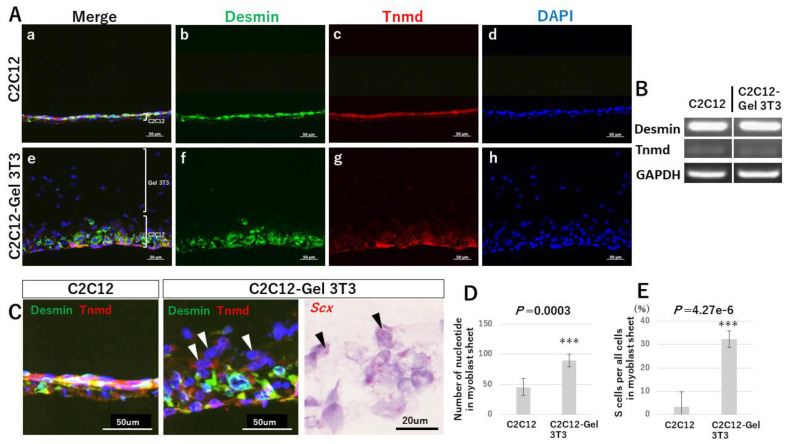
Morphology of myoblast sheets of C2C12-Gel 3T3, in comparison with C2C12 after 1 week of culture. (**Panels A**,**D**) The total cell count in the myoblast sheets was greater in C2C12-Gel 3T3 than in C2C12 (C2C12: 45.67 ± 14.30, C2C12-Gel 3T3: 89.83 ± 10.79, *n* = 6). Desmin and Tnmd were expressed. No difference in desmin and Tnmd expression was evident between C2C12 and C2C12-Gel 3T3 (Panel **A b**,**c**,**f**,**g**). (**Panel B**) mRNAs for desmin and Tnmd were expressed in the cell sheets. (**Panels C**,**E**) The boundary between C2C12 and the gel in C2C12-Gel 3T3. Desmin was not expressed, but superficial cells expressing Tnmd (S cells, arrowheads) were evident. However, few S cells were identified in C2C12 (C2C12: 3.50 ± 6.21%, C2C12-Gel 3T3: 32.27 ± 3.59%, *n* = 6). The S cells expressed Scx. *** *p* < 0.001.

We examined the morphology of C2C12-Gel 3T3 myoblast sheets in comparison with C2C12. The total cell count in the myoblast sheets was significantly greater for C2C12-Gel 3T3 than for C2C12 (*p* = 0.0003) (Figure 4A,D). Therefore, cell sheet thickness was increased to a greater degree in C2C12-Gel 3T3 than C2C12. Immunohistochemistry for desmin (a myoblast marker) and Tnmd (expressed comparatively later in tendon development) was used (Figure 4A(b,c,f,g)). No difference in desmin or Tnmd expression was observed between C2C12 and C2C12-Gel 3T3 (Figure 4A(b,c,f,g)). Observation of mRNA expression in C2C12 and C2C12-Gel 3T3 revealed that mRNAs for desmin and Tnmd were expressed in the cell sheets (Figure 4B). Expression of Tnmd mRNA was weak (Figure 4B). Next, focusing on the boundary between C2C12 and the gel in C2C12-Gel 3T3, we found that desmin was not expressed, but superficial cells expressing Tnmd (S cells) were observed (Figure 4C, arrowheads). However, few S cells were identified in C2C12 (Figure 4C). The number of S cells was significantly larger in C2C12-Gel 3T3 relative to C2C12 (C2C12 vs. C2C12-Gel 3T3; *p* = 4.27 × 10^−6^) (Figure 4E). Moreover, S cells expressed scleraxis (Scx), a tendon progenitor marker (Figure 4C).

### 2.5. Effects of Myostatin Addition on the Pseudo MTJ Model

**Figure 5:**
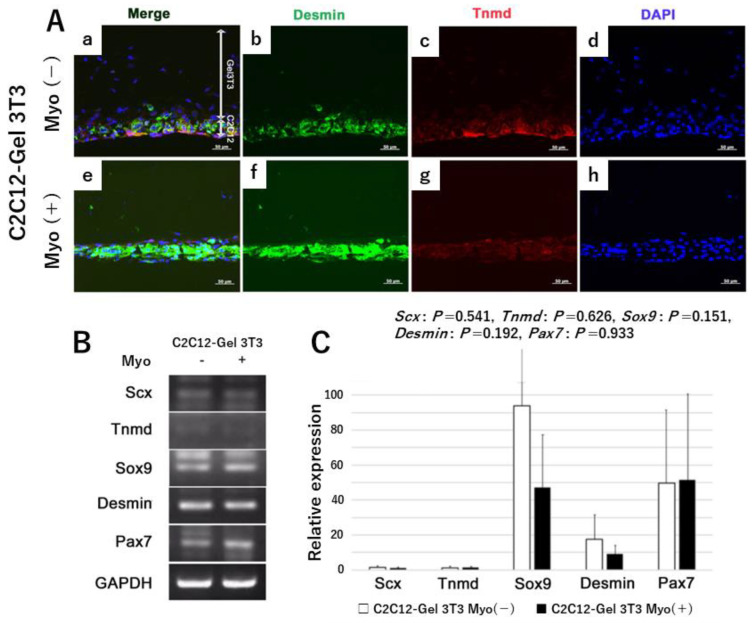
Expression of related proteins and genes in C2C12-Gel 3T3 sheets after 1 week of culture. (**Panel A**) (**a**–**h**) Immunohistochemical staining of C2C12-Gel 3T3. The upper row shows the Myo-free group (Myo(−)), while the lower row shows the Myo-added group (Myo(+)). Desmin and tenomodulin (Tnmd) were expressed in all myoblast sheets (Panels b,c,f,g). Dispersion of cell nuclei revealed by DAPI staining: in comparison to the Myo(−) group, nuclei were regularly aligned in the Myo(+) group (Panels d,h). (**Panels B**,**C**) Expression of related genes Scx, Tnmd, Sox9, desmin, and Pax7: no difference in expression level was evident between Myo(−) and Myo(+) (*n* = 4).

To clarify the function of Myo underlying the formation of the MTJ, we compared C2C12-Gel 3T3 sheets with (Myo(+)) and without (Myo(−)) (Figure 5A). Regarding the Immunohistochemistry for desmin and Tnmd (Figure 5A(b,c,f,g)), no difference in desmin or Tnmd expression was observed between Myo(−) and Myo(+) sheets (Figure 5A(b,c,f,g)). Cell dispersion was larger in Myo(−) than in Myo(+) sheets (Figure 5A(d,h)). We identified mRNA expression of related genes in C2C12-Gel 3T3. The target genes were Scx, Tnmd, Desmin, Sox9 (a transcription factor common to tendon, muscle, and bone development), and Pax7 (a transcription factor of satellite cells). Expression levels of all mRNAs in the cell sheets showed no significant difference between Myo(−) and Myo(+) (Figure 5B,C) (*Scx*: *p* = 0.541, *Tnmd*: *p* = 0.626, *Sox9*: *p* = 0.151, *Desmin*: *p* = 0.192, *Pax7*: *p* = 0.933).

**Figure 6:**
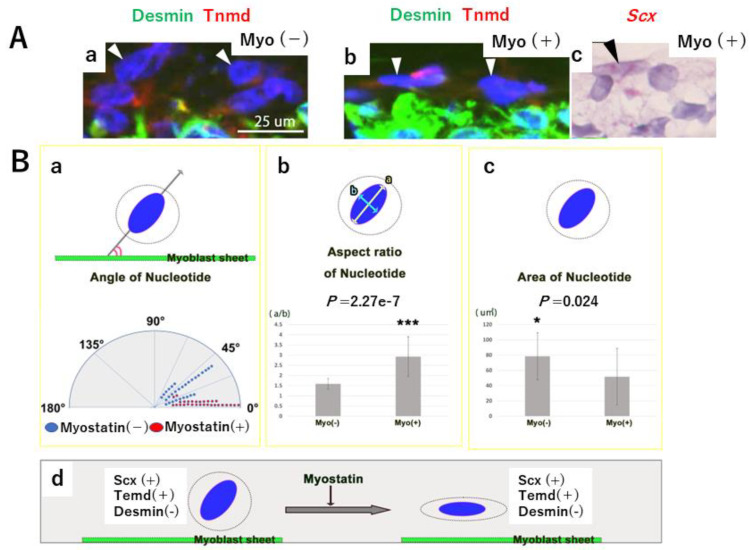
Boundary between C2C12 and gel in C2C12-Gel 3T3 after 1 week of culture. (**Panel A**) Morphology of S cells (white arrows) at the boundary between C2C12 and the gel in C2C12-Gel 3T3 (Panel **a**: Myo(−), Panels **b**,**c**: Myo(+)). S cells in Myo(+) expressed Scx (Panel c). (**Panel B**) The angle between the cell sheet is significantly smaller in Myo(+) than in Myo(−) cultures (Myo(−): 28.53 ± 17.22°, Myo(+): 12.13 ± 5.13°) (Panel **a**). The long axis of the cell nucleus is significantly smaller in Myo(−) than in Myo(+) cultures (Myo(-): 1.52 ± 0.23, Myo(+): 2.98 ± 1.02) (Panel **b**). The cross-sectional area of the nuclei of S cells in Myo(+) and Myo(−) cultures is significantly smaller in Myo(+) than in Myo(−) cultures (Panel **c**) (Myo(−): 79.87 ± 30.5 µm^2^, Myo(+): 53.23 ± 32.23 µm**^2^**). Ten S cell nuclei are randomly selected from each of the three Myo(−) and Myo(+) samples (10 nuclei × 3 samples = 30 nuclei). S cell nuclear morphology in Myo(+) and Myo(−) cultures (Panel **d**). * *p* < 0.05 and *** *p* < 0.001.

We then focused on S cells at the boundary (i.e., the “pseudo MTJ”) between C2C12 and the gel in C2C12-Gel 3T3 (Figure 6A). S cells in Myo(+) cultures expressed Scx to a similar degree as those in Myo(−) cultures (Figure 4C and Figure 6A(c)). The angle between the cell sheet and the long axis of the cell nucleus was significantly smaller in Myo(+) than in Myo(−) cultures (*p* = 0.0003) (Figure 6A,B(a)). This showed that in Myo(+), S cells were oriented horizontally on the surface of the myoblast sheets in C2C12-Gel 3T3. In addition, the aspect ratio of the long axis to the horizontal axis of S cell nuclei was significantly larger in Myo(+) than in Myo(−) (*p* = 2.27 × 10^−7^) (Figure 6A,B(b)). Therefore, compared to Myo(−) cultures, the nuclei of S cells in Myo(+) cultures were spindle-shaped. Investigation of the cross-sectional area of S cell nuclei showed that it was significantly smaller in Myo(+) than in Myo(−) cultures (*p* = 0.024) (Figure 6A,B(c)). Accordingly, the nuclei of S cells at the boundary between the myoblast sheet and the gel exhibited a morphology close to a circle in Myo(−) cultures, and a spindle shape in Myo(+) cultures (Figure 6B(d)), suggesting that the nuclei of S cells changed their morphology in response to Myo. This result was similar to that obtained in vivo, indicating that Myo changes the morphology of the nucleus to a spindle-like shape in cells differentiating on the tendon side of the MTJ.

## 3. Discussion

In the present study, we investigated the role of Myo in promotion of MTJ regeneration using a prepared model of Achilles tendon injury and laminated cell sheets, adopting both an in vivo and an in vitro approach. It is well known that Myo inhibits muscle differentiation and growth, and recently it has also been shown to play a positive role in tendon maintenance [14,15]. Here we found that Myo changed the nuclear morphology of tendon progenitor cells adjacent to the MTJ. Ho et al. [31] demonstrated a direct correlation between the aspect ratio of the cell and that of the nucleus. Moreover, the nucleus determines changes in cellular shape for proprioception to control dynamic behavior [32]. In other words, Myo changes the morphology of tendon progenitor cells close to the MTJ. However, it is unknown whether Myo directly affects cell or nuclear morphology.

Co-culture, in which two or three cell types are cultured together, is an in vitro method for investigation of cell–cell interaction and communication, which influence cellular proliferation, differentiation, and maturation [33]. Shima et al. [34] have reported that NIH3T3 fibroblast cells encapsulated in cell fibers (a technique for three-dimensional cell culture) promoted the differentiation of C2C12 myoblasts. Koeck et al. [27] found that collagen fibers with good mechanical stability assisted the differentiation of C2C12 myoblasts into myotubes showing morphologic alignment along the fiber axis. Moreover, they developed an MTJ model by co-culturing C2C12 and NIH3T3 on a collagen fiber scaffold. In the present study, we laminated NIH3T3 fibroblasts on C2C12 myoblasts, and found that in this co-culture model (C2C12-Gel 3T3) the fibroblasts promoted proliferation of the myoblasts. Since Scx was expressed in S cells, these cells were regarded as tendon progenitor cells. Therefore, our C2C12-Gel 3T3 co-culture model was able to represent the boundary between muscle and tendon, i.e., the MTJ. Although the C2C212 and NIH3T3 cells were not aligned in this model, it is considered to be a useful and simple approach for investigation of muscle–tendon communication.

Recently, Yaseen et al. [10] demonstrated that fibroblasts undergo switching to a myogenic fate at the MTJ. These dual-identity fibroblastic cells then fuse into the developing muscle cells adjacent to the MTJ. However, it is unclear whether myoblasts undergo switching to a fibroblast or a tendon fate. Although our study did not investigate the origin of S cells, it is considered that they must have been derived from either C2C12 or NIH3T3 cells.

In previous tissue regeneration studies involving cell sheet engineering, we had investigated various in vivo phenomena under in vitro conditions [28,29]. Yamane et al. [28] succeeded in creating three-layered hybrid cell sheets similar to the structure of the oral mucosa, including submucosal muscles using oral mucosal epithelial cells, mesenchymal cells, and myoblasts from rabbits. Each cell type was obtained from the oral mucosa using enzymatic digestion. In addition, isolated epithelial cells were cultured for 2 weeks on collagen containing isolated mesenchymal cells, and then laminated to myoblast sheets. Umezawa et al. had previously demonstrated enhanced proliferative activity of skeletal myoblasts in the presence of mesenchymal stem cells [29].

As described in the Introduction, Myo is known to be a factor that regulates muscle growth [12,13]. Bright-field observations, illustrated in Panel B of Figure 3, showed that in cultures where Myo was not added to C2C12-Gel 3T3, myoblast differentiation advanced, and many cells exhibited myotube-like structures. However, in cultures treated with Myo, cells exhibiting myotube-like structure were rarely observed. In addition, observation of longitudinal sections, illustrated in Panel C of Figure 3, showed that the thickness of myoblast sheets increased in the absence of Myo, but rarely changed in cultures where Myo was added to C2C12-Gel 3T3. This suggested that Myo regulates the differentiation and maturation of myoblast sheets, thus supporting previous reports on the effects of Myo on muscle development and growth. We then investigated whether or not differentiation was promoted in C2C12-Gel 3T3 by examining the expression of several related genes at the mRNA level. As we speculated that the differentiation and maturation of myoblast sheets would be affected by signals from NIH3T3 in the gel, we examined not only markers of muscle development, but also markers of tendon development. Expression of Pax7 and desmin, as well as Sox9, was detected. Tnmd was also detected at the mRNA level. Although Sox9 is known to be a marker of cartilage and bone development, in recent years it has also been reported to be a marker common to the “muscle–tendon–bone complex” at muscle attachment sites [35,36,37,38,39]. A paper examining the origin of the extraocular muscles that move the eyeball shows that several muscles originate from common tendons for their complex functions [40]. In this way, especially in the oral region, masticatory function is related to the morphology of soft tissues and pore tissues. For example, in a paper on experimental tooth loss, it was shown that the morphology and constituent proteins of the oral mucosa changed [41]. However, Tnmd was also detected by immunohistochemistry; Tnmd-positive cells were observed lining the base of myoblast sheets and at sites in contact with the gel (Figure 3C). Although myoblasts were induced into tendons, it remains unclear whether this resulted from the effect of NIH3T3 or Myo.

Scx, a transcription factor required for tendon differentiation, is a known marker of tendon development expressed in tendon progenitor cells [42,43,44,45]. The musculoskeletal system comprises muscles, tendons, and skeletal system linked at their junctions, functioning in a cooperative manner as the means of locomotion. Using ScxCre knock-in mice, Yoshimoto et al. [45] have demonstrated that Scx, a transcription factor expressed in tissues connecting the musculoskeletal system, is required for not only tendons and ligaments—where its expression is long-lasting—but also the maturation of cartilage at the junctions, where it is expressed only transiently. It is expected that the relationship between Mkx, a transcription factor required at the middle stage of tendon development [46], and Scx will become an important focus of future research. Berthet et al. [46] have investigated Smad3, a downstream effector of TGFβ, which plays an important role in the formation and healing of tendons. They were able to show that, in Smad3-deficient (Smad3−/−) mice, expression of collagen I appeared to decrease and tendon structure was destroyed. This not only affected tendon development but also led to fragility of the tendon structure in adult mice. We speculated that Smad3 might affect Scx and Mohawk, which are important tendon transcription factors. However, few previous studies have focused on development of the MTJ, and some unclear points remain. In the present study, we found that many cells at the boundary between the myoblast sheet and the gel differentiated into tenocytes; in addition, their morphology changed from round to flat following addition of Myo (Figure 6). As this was also observed in the MTJ during regeneration following injury, it appears that Myo is able to change the morphology of cell groups connected to other tissues. When tendon tissue was experimentally destroyed at the center of the Achilles tendon, the histological appearance of the MTJ also suggested fragility (Figure 1). At that point, Myo was found to be expressed on the muscle side of the MTJ and increased over time.

Here we have shown that Myo affected the regeneration of tendon tissues in contact with muscle, and morphological changes evident in vivo were similar to those observed in vitro. These changes suggest a phenomenon by which the connectivity of the MTJ is strengthened. The present findings are, however, preliminary, and further studies will be needed to clarify the mechanisms mediating the morphogenesis and maintenance of the MTJ in vivo.

## 4. Materials and Methods

### 4.1. Achilles Tendon Injury Model

All animal experiments were conducted in accordance with the National Institutes of Health guidelines for care and use of animals. In addition, this study was conducted with the approval of the Tokyo Dental College Institutional Animal Care and Use Committee protocol #210106). A mixture of medetomidine (0.3 mg/kg), midazolam (4.0 mg/kg), and butorphanol (5.0 mg/kg) was administered intraperitoneally to 6-week-old C57BL6J mice for deep anesthesia. An incision about 2 cm long was made on the skin of both hind legs, then the Achilles tendon was exposed and completely transected [47]. The mice were euthanatized with CO_2_ on postoperative days 7 and 28 (POD7 and POD28), and the Achilles tendon and gastrocnemius muscle connected to it were collected en bloc. After collection, most muscle tissues were removed from the Achilles tendon under a microscope (Stemi305; Carl Zeiss Inc., NY, USA) until the muscle side of the MTJ and tendon tissue remained. Six specimens were fixed with 4% paraformaldehyde phosphate (PFA) and then paraffin-embedded using the standard method. Subsequently, 5 μm thick serial tissue sections were prepared. The nuclear morphology on the tendon side of the MTJ was assessed after staining with hematoxylin and eosin (H-E) (NIH, USA, http://rsbweb.nih.gov/ij/ accessed on 5 August 2022.). Nuclei within four 100 μm square areas were analyzed on the tendon side of the MTJ. Ten samples each of ① extramuscular and ② extramuscular + intramuscular tendons + the muscle side of the MTJ were prepared, and the expression of Myo mRNA was compared at POD7 and POD28 (Figure 1). The tendon connected to the gastrocnemius is divided into two parts: the proximal (intramuscular) tendon and the distal tendon. In Figure 1, stars indicate the boundary of the two. We observed unexpected cell accumulation at the boundary (Figure 1C). If we had clearly divided 1 into 2, some tissues may not have included cell accumulation. Therefore, we compared ① to ① + ②. Table 1 shows the details of the primer assay ID and amplicon length for real-time PCR.

### 4.2. Preparation of the Mouse Myoblast, Mesenchymal Cell Line

C2C12 cells and NIH3T3 cells were cultured in Advanced Dulbecco’s modified Eagle medium (A-DMEM) supplemented with 10% fetal bovine serum (FBS). After 2 weeks at 37 °C in humidified air with 5% CO_2_, the cells were passaged before reaching confluence.

### 4.3. Preparation of Cell Sheets and Culture Conditions for the Pseudo MTJ Model

C2C12 cells were placed in the cell culture inserts of 6-well plates (Transwell; Corning, NY, USA) at a density of 1.0 × 10^4^ cells. In the next step, the following three different cultures were then prepared and placed in the inserts to investigate the effects of co-culture with NIH3T3 on the myoblast sheets (Figure 1):

(1) Myoblast sheet only (as a control) (C2C12);
(2) A layer of collagen gel (Cellmatrix^Ⓡ^; Nitta-gelatin Co., Osaka, Japan) on top of myoblasts (C2C12-Gel);
(3) A well-stirred mixture of collagen gel and NIH3T3 cells overlaid on myoblasts (C2C12-Gel 3T3),

NIH3T3 cells were seeded at a density of 2.5 × 10^4^ cells/800μL collagen gel/well. In this in vitro model, the MTJ was assumed to be an area of contact between the myoblast sheet and overlaid collagen gel with NIH3T3 (Figure 1, arrowhead). Superficial cells of the myoblast sheet present in the MTJ region were named “S cells” (Figure 1, arrows). To compare the effect of tenocyte differentiation in each condition, cell sheets were cultivated in 2 mL A-DMEM culture media containing 10% FBS with/without GDF-8 (500 ng/mL Myo, mouse, recombinant, R&D Systems). The volume of medium above and below the culture layer was 0.5 mL and 1.5 mL, respectively. The cell sheets were harvested after 1 week of culture to determine the progress of cell sheet development.

### 4.4. Histochemical Analysis (In Vitro)

To prepare samples for histochemical staining, two pieces (10 × 0.5 mm) were excised from the harvested cell sheets and embedded in Tissue-Tek compound. Frozen 5 μm sections were prepared and subjected to hematoxylin–eosin (H-E) staining and immunohistochemical (IHC) staining for histological observation. For the IHC staining, the section was fixed with 2% paraformaldehyde for 5 min and incubated with blocking solution (10% normal donkey serum and 1% BSA in 0.001 M PBS) for 60 min. The primary antibodies comprised anti-desmin antibody (1/300, D9, LSBio, and LS-B7175) and anti-tenomodulin antibody (1/100, LSBio, LS-B8193). The sections were incubated with the primary antibodies at room temperature for 90 min. For fluorescence labeling, the sections were incubated with a secondary antibody, comprising either Cy3-donkey anti-mouse IgG (CHEMICON, AP192C) or Rho-donkey anti-mouse IgG (Jackson, 715-025-150) at room temperature (RT) for 30 min.

Cell nuclei were stained with 0.5 mg/mL 4′,6-diamino- 2-phenylindole (DAPI; Dojindo Laboratories, Tokyo, Japan) for 5 min. The stained sections were viewed using a fluorescence microscope (Axioplan2 imaging; Carl Zeiss Inc., NY, USA). In addition, the nuclear morphology of the S cells observed at the boundary between C2C12 and the gel in C2C12-Gel3T3 was assessed in DAPI-stained images (NIH, USA, http://rsbweb.nih.gov/ij/ accessed on 5 August 2022.). We defined superficial cells in the myoblast sheet as S cells. The cells expressed Scx and Tnmd, but not desmin. However, we did not investigate whether the cells originated from C2C12 or NIH3T3. Ten S cell nuclei were randomly selected from each of the three Myo(−) and Myo(+) samples (10 nuclei × 3 samples = 30 nuclei). The measured items were as follows: ① angle between cell sheet and nucleus, ② aspect ratio of the nucleus, and ③ nucleus area (Figure 6B).

### 4.5. In Situ Hybridization

To evaluate undifferentiated tenocytes in cell sheets, we performed in situ hybridization for scleraxis (Scx) (a marker of tendon progenitors). Scx anti-sense probes reported previously were used [48]. The probes were labeled with digoxigenin (DIG RNA labeling mix; Roche, Rotkreuz, Switzerland), and hybridization was performed according to the standard method. Fixation with 4% paraformaldehyde was performed for 10 min, followed by a 5 min degradation with 1 μg/mL proteinase K (Roche), and a second fixation for 5 min. Next, acetylation was performed for 10 min with a solution containing triethanolamine, hydrochloric acid, and acetic anhydride. Sections were pre-blocked with hybridization buffer (50% formamide, 5× saline sodium citrate [SSC], 50 μg/mL yeast tRNA, 1% sodium dodecyl sulfate, and 50 μg/mL heparin), and incubated with the Scx anti-sense probes diluted to 1 ng/μL with hybridization buffer. After washing off unbound probes using saline sodium citrate buffer, the probes on the sections were detected using anti-digoxigenin antibodies bound to ALP (Roche) and BM Purple (Roche). Each section was observed using an upright microscope (Axio imager2; Carl Zeiss Inc., Oberkochen, germany. NY, USA).

### 4.6. Reverse Transcription-Polymerase Chain Reaction

The remaining cell sheets were collected for RNA purification. RNA was extracted using the SV Total RNA Isolation System (Promega, WI, USA) and cDNA synthesized with avian myeloblastosis virus reverse transcriptase (Takara Bio Inc., Shiga, Japan). To investigate mRNA expression for genes related to myoblasts, reverse transcription-polymerase chain reaction (RT-PCR) analysis was conducted for those associated with maintenance of the undifferentiated state in myoblasts (desmin and Pax7), tenocytes (Scx, Tnmd), and a transcription factor common to muscle, tendon, and bone development (Sox9), in addition to the genes encoding desmin and Tnmd that had been investigated using IHC staining. Glyceraldehyde-3-phosphate dehydrogenase (GAPDH) was used as an internal standard. RT-PCR cycling conditions included 30 cycles consisting of thermal denaturation at 95 °C for 30 s, annealing at 52 °C for 30 s, and extension at 72 °C for 20 s, followed by a final extension at 72 °C for 5 min. Gel electrophoresis was carried out at 100 V for 20 min. Primer sequences and product sizes for the RT-PCR and primer assay ID and amplicon length for real-time PCR are shown in Table 1 and Table 2.

### 4.7. Statistical Analysis

All statistical analyses were performed using SPSS 21.0 (IBM, Armonk, NY, USA). Statistical tests, *n* values, replicate experiments, and *p* values are all located in the figures and/or legends. All data are presented as the mean  ±  S.D. *p* values were calculated using Student’s *t*-test and Mann–Whitney U test (* *p* < 0.05, ** *p* < 0.01, and *** *p* < 0.001 are used throughout the paper).

## 5. Conclusions

Here, we laminated collagen gel mixed with NIH3T3 to C2C12 cells and observed changes in the laminated sheets after addition of Myo to clarify its role in the MTJ in vitro. The present results indicate that Myo promotes the maturation of cells at the boundary between the myoblast sheet and the gel, helping to strengthen the connectivity of the MTJ at an early stage of growth and wound healing. However, the present study did not clearly demonstrate the regulatory involvement of Myo in vivo. Further studies to investigate the role of Myo at the MTJ will be necessary.

## Figures and Tables

**Table 1:** Primer assay ID and amplicon length for real-time PCR.

Primer		Assay ID	Amplicon Length
Scleraxis	transcription factor for differentiated tendon cells.	Mm01205675_m1	59
Tenomodulin	late differentiation marker gene for tendon and ligament tissue.	Mm00491594_m1	65
Sex determining region Y-box9	transcription factor for differentiated chondrocyte cells and tendon cells.	Mm00448840_m1	101
Desmin	Type III intermediate filament in skeletal, smooth and cardiac muscle tissue.	Mm00802455_m1	92
Paired Box 7	Paired box transcription factor family member involved in maintaining proliferation and preventing differentiation in skeletal muscle progenitor cells.	Mm00482759_m1	102
Myostatin	Negative regulated protein for muscle	Mm01254559_m1	90
S18	S13P family of ribosomal proteins used as an internal control.	Mm02601777_g1	76

**Table 2:** Primer sequences and product sizes for RT-PCR.

Primer		Sequence (5′–3′)	Product Size (bp)
Scleraxis	transcription factor for differentiated tendon cells.	AGCCCAAACAGATCTGCACCTT	139
		CTTCCACCTTCACTAGTGGCATCA	
Tenomodulin	late differentiation marker gene for tendon and ligament tissue.	ATGGGTGGTCCCACAAGTGAA	123
		CTCTCATCCAGCATGGGATCAA	
Sex determining region Y-box9	transcription factor for differentiated chondrocyte cells and tendon cells.	ATCTGAAGAAGGAGAGCGAG	263
		TCAGAAGTCTCCAGAGCTTG	
Desmin	Type III intermediate filament in skeletal, smooth and cardiac muscle tissue.	ACCAGATCCAGTCCTACACC	202
		TTGAGCAGGTCCTGGTACTC	
Paired Box 7	Paired box transcription factor family member involved in maintaining proliferation and preventing differentiation in skeletal muscle progenitor cells.	ATCCGGCCCTGTGTCATCTC	278
		CACGCGGCTAATCGAACTCA	
Glyceraldehyde 3-phosphate dehydrogenase	Glyceraldehyde-3-phosphate dehydrogenase, internal control.	ACCACAGTCCATGCCATCAC	452
		TCCACCACCCTGTTGCTGTA	

## Data Availability

Not applicable.
